# Specialty grand challenge in gastrointestinal infections

**DOI:** 10.3389/fgstr.2026.1808344

**Published:** 2026-03-02

**Authors:** Sahil Khanna

**Affiliations:** Gastrointestinal Infection Section, Frontiers in Gastroenterology, Mayo Clinic, Rochester, MN, United States

**Keywords:** antimicrobial resistance, antimicrobial stewardship, diagnostic innovation, gastrointestinal infections, microbiome-based therapeutics

## Introduction

Gastrointestinal (GI) infections remain a leading cause of morbidity worldwide, spanning self−limited acute gastroenteritis to life−threatening colitis, enteric fever, and healthcare-associated outbreaks. Recent Global Burden of Disease (GBD analyses continue to rank diarrheal diseases among the highest contributors to global illness; one GBD 2021 analysis reported ~4.67 billion incident cases in 2021 and estimated diarrheal deaths declining from ~1.26 million in 2019 to ~1.17 million in 2021 (1). There has been real but uneven progress as risk and outcomes are shaped by access to safe water and sanitation, nutrition, healthcare infrastructure, and the growing rates of immunosuppression and comorbid conditions (2). Antimicrobial resistance (AMR) is limiting therapeutic options for many enteric pathogens and amplifying harms, with global analyses estimating substantial mortality attributable to bacterial AMR (3).

The epidemiology of GI infections continues to be dynamic. Rotavirus remains an important contributor to severe childhood diarrhea globally with vaccine introduction markedly reducing deaths where coverage and uptake is high (4). Norovirus is widely recognized as the most common cause of acute gastroenteritis globally and a major driver of outbreaks across community and healthcare settings (5). In high-income regions, *Clostridioides difficile* infection (CDI) has emerged as a defining challenge of modern infection prevention, with substantial incidence and severe outcomes in the hospital and the community (6). Contemporary CDI guidelines reflect an evolving therapeutic landscape that includes narrow-spectrum microbiome-sparing antibiotics for its treatment and adjunctive and microbiota-based recurrence prevention strategies (7, 8).

The Gastrointestinal Infection section of Frontiers in Gastroenterology aims to catalyze progress across epidemiology, diagnostics, therapeutics, infection prevention, antimicrobial stewardship, and the microbiome. This Specialty Grand Challenge highlights key problems and priorities for research and practice over the next decade, emphasizing translation into equitable impact.

## Epidemiological challenges

### Shifting burden across age, geography, and settings

Global analyses underscore that diarrheal mortality remains substantial and unevenly distributed. The pandemic era underscored both the responsiveness of transmission to public health measures and the persistence of structural inequities; one GBD 2021 analysis reported adults older than 70 years as the highest-risk age group globally during 1990–2021 trend analyses, surpassing under−five children in deaths per population in 2021 (1). In parallel, etiologies vary sharply by socio-demographic context: rotavirus and cryptosporidiosis remain dominant contributors in lower−resource settings, while CDI is disproportionately important in high−SDI regions (1, 2).

In high-income settings, the challenge is often less about incidence and more about complexity: immunomodulators and oncology therapies, advanced endoscopy and surgery, and prolonged post-acute care create new opportunities for enteric pathogens to cause severe disease, spread within healthcare networks, and interact with the host microbiome. In low-resource settings, the challenge remains the persistent convergence of enteric pathogens with undernutrition, limited access to diagnostics, delayed care, and Water, Sanitation, and Hygiene (WASH) deficits, which magnify both incidence and severity (2).

### The “new” epidemiology driven by diagnostics

Culture−independent diagnostic tests (CIDTs) and multiplex gastrointestinal panels have reshaped surveillance and perceived incidence for several pathogens. The increasing use of syndromic GI panels has, for example, changed understanding of yersiniosis epidemiology in the United States, with more diagnoses in adults and different seasonality than previously appreciated (9). It has also increased the proportion of falsely abnormal tests.

However, CIDT adoption can paradoxically weaken public health response when reflex culture is incomplete. Public health laboratories report lower recovery of enteric bacteria from CIDT-positive specimens than from culture-derived isolates, increasing costs and limiting the isolates needed for outbreak detection, molecular subtyping, and antimicrobial susceptibility testing (10). This creates a surveillance gap precisely when pathogen evolution is accelerating.

### Drivers of emergence: mobility, inequity, and vulnerable hosts

International travel, globalized food systems and population displacement can amplify transmission and introduce resistant lineages into new settings (11, 12). Meanwhile, vulnerable host populations are expanding: older adults, transplant recipients, patients receiving immunosuppression, biologics or chemotherapy, and people with chronic liver disease or inflammatory bowel disease. These trends mean that epidemiology must be studied not only in populations, but within interconnected “ecosystems” of community, hospital, long-term care, and the environment.

A grand challenge for the field is to build surveillance that is faster, more granular, and more equitable integrating clinical data, laboratory analytics, genomics, and environmental signals while also protecting privacy and enabling rapid, practical public health action.

## Diagnostic challenges

### The promise and pitfalls of syndromic molecular testing

Multiplex PCR panels can identify pathogens rapidly and may support earlier optimization or de−escalation of antibiotics and infection control measures. Implementation of the BioFire FilmArray GI panel, for instance, has been associated with reductions in antibiotic use and earlier transition to optimal therapy in some hospital settings (13). Yet syndromic panels introduce new interpretation problems. Co-detections are common, and panels may detect colonization, low-level carriage, or prolonged shedding, particularly in children and immunocompromised hosts (12). Without quantitative outputs, clinicians often lack a principled way to distinguish causation from coincidence. The resulting uncertainty can drive both over-treatment (unnecessary antibiotics) and under-treatment (discounting actionable findings).

### CDI testing: distinguishing infection from colonization

CDI is a diagnostic “stress test” for modern laboratory medicine. Highly sensitive nucleic acid amplification tests like PCR assays can over-diagnose CDI if used without appropriate clinical criteria or confirmatory toxin testing. Both gastroenterology and infectious diseases guidelines emphasize diagnostic algorithms that incorporate a sensitive and a specific test (or careful clinical pretest probability) to distinguish colonization from toxin-mediated disease (7, 8).

Diagnostic misclassification has downstream consequences: inappropriate isolation and antibiotics, inflated hospital metrics, increased costs, and missed alternative diagnoses (e.g., medication-related diarrhea, IBD flare, ischemic colitis). CDI diagnostics therefore exemplify the broader need for diagnostic stewardship: testing only in appropriate clinical contexts, using algorithms aligned with the local laboratory’s strengths, and reporting results with interpretive guidance.

### Restoring lost capabilities: culture, susceptibility, and public health action

As CIDTs replace culture, the field risks losing routine access to phenotypic antimicrobial susceptibility and the isolates needed for outbreak detection. Lower recovery of pathogens from CIDT-positive specimens undermines surveillance and drives up public health laboratory costs (10). A practical path forward will involve tiered diagnostics: syndromic panels for rapid clinical decisions; selective reflex culture for specific targets, severe disease, outbreak signals, or when susceptibility is clinically important; and integration of genomic or metagenomic approaches to recapture typing and resistance information.

### Metagenomics and host-response diagnostics: toward next-generation precision

Shotgun metagenomics is increasingly feasible for enteric pathogen detection. Studies demonstrate high sensitivity and specificity for common bacterial pathogens in stool, and emphasize the potential to unify diagnosis with surveillance by enabling detection plus genomic characterization in a single workflow (14). More recent work suggests metagenomics may be applied as a routine pathology test for GI infections, potentially expanding the range of detectable agents without target bias (15).

Challenges that remain include cost, turnaround time, sample preparation, contamination control, bioinformatic standardization, and most importantly clinical interpretation. Parallel progress in host-response diagnostics (e.g., multi-omics signatures of invasive bacterial infection) could help assign causality and guide therapy, but will require rigorous validation across age groups, geographies, and immunologic states.

## Therapeutic challenges

### Supportive care remains foundational—but implementation is uneven

For most acute infectious diarrhea, optimal management hinges on hydration and electrolyte replacement. The IDSA guidelines underscore the centrality of supportive care and careful risk stratification, especially for infants, older adults, and immunocompromised patients (12). However, implementation remains uneven, particularly where access to oral rehydration solution, diagnostics, and timely referral is limited.

### Antimicrobial therapy in an era of resistance and collateral damage

When antibiotics are indicated, choices are increasingly constrained by resistance and by collateral harms: selection for multidrug-resistant organisms, microbiome disruption, and increased CDI risk. In travel-associated diarrhea, CDC guidance discourages prophylactic antibiotics for most travelers and highlights that antibiotic exposure increases the risk of acquiring resistant Enterobacterales; when treatment is needed, therapy should be matched to syndrome severity and likely pathogens.

A continuing grand challenge is building evidence that supports targeted therapy: who benefits from antibiotics, which agents remain effective in a given region, and what duration minimizes harms while preserving outcomes. This requires harmonized, practical endpoints (including patient-reported outcomes and post-infectious sequelae), and better integration of resistance surveillance with clinical guidance.

### CDI: recurrences, microbiome repair, and durable prevention

CDI illustrates the dilemma of treating a dysbiosis-driven infection with antibiotics that further perturb the microbiome. The 2021 focused update from IDSA/SHEA prioritizes fidaxomicin when feasible (7). Microbiota-based approaches have matured from traditional fecal microbiota transplantation (FMT) to standardized, regulated products supported by randomized trials. Fecal microbiota spores, live-brpk (an oral microbiome therapy) reduced recurrence in a randomized trial (16), and fecal microbiota, live-jslm demonstrated superiority over placebo in a phase III trial using a Bayesian primary analysis (17). In the United States, the FDA has approved these two microbiota-based products for prevention of recurrent CDI after antibacterial treatment (18, 19).

### *Helicobacter pylori*: resistance-driven regimen selection and the need for susceptibility testing

Eradication of *Helicobacter pylori* remains central to ulcer disease management and gastric cancer prevention, but rising resistance has eroded traditional clarithromycin- and fluoroquinolone-based regimens. The Maastricht VI/Florence consensus and the 2024 ACG guideline both emphasize empiric bismuth quadruple therapy when susceptibility is unknown, the importance of test-of-cure, and limiting clarithromycin- or levofloxacin-containing salvage regimens to cases with documented susceptibility (20, 21).

The therapeutic challenge is implementing susceptibility-guided care at scale. That includes expanding access to molecular resistance testing, validating point-of-care and noninvasive sampling approaches, and ensuring that new acid-suppressing strategies and novel antibiotics are evaluated with outcomes that matter (eradication, adverse events, cost, and long-term cancer prevention).

### The unmet need: antivirals, antiparasitics, and new anti-infectives

For norovirus and many viral gastroenteritides, specific antivirals remain limited, making prevention and outbreak control paramount (5). For protozoa such as Cryptosporidium, treatment options are limited in immunocompromised hosts, and drug development has lagged. Novel anti-infectives, monoclonal antibodies, bacteriophages, and anti-virulence strategies are promising, but translation requires better trial infrastructure, harmonized endpoints, and integration with stewardship to avoid repeating the cycle of resistance.

## Challenges in infection prevention

### Community prevention: vaccines, WASH, and food safety

The most durable reductions in GI infection burden come from prevention: clean water, sanitation, hygiene, safe food systems, and vaccination. Rotavirus vaccination has reduced mortality and morbidity, with global analyses estimating tens of thousands of deaths averted each year and many more preventable with full coverage (4).

Norovirus prevention is an active frontier. The WHO highlights the large global burden and supports vaccine research that could reduce both sporadic disease and outbreaks in healthcare and congregate settings (5). For enteric fever, cholera, and other vaccine-preventable enteric infections, the challenge is implementing vaccines alongside WASH and food safety interventions to achieve durable, equitable impact.

### Healthcare prevention: CDI as a model for transmission control

CDI prevention depends on environmental decontamination, hand hygiene, contact precautions, judicious antibiotic and acid-suppressing medication use, and surveillance across care transitions. National estimates highlight the substantial U.S. burden of CDI despite prevention efforts (6). Because healthcare is increasingly distributed across hospitals, long-term care, outpatient infusion, and home health, infection prevention must be network-aware: interventions should target transmission pathways across facilities, not just within a single ward.

### Safety of microbiota-based interventions

Microbiota-based strategies carry unique safety considerations. FDA safety communications have highlighted the risk of transmitting multidrug-resistant organisms and other pathogens via FMT and have outlined donor screening and testing requirements, including during the COVID−19 pandemic (22).

The grand challenge is to preserve the therapeutic promise of microbiome repair while making safety systematic and scalable. Standardized manufacturing, validated pathogen screening, robust pharmacovigilance, and long-term follow-up are essential, particularly as microbiota-based products expand beyond recurrent CDI into broader indications.

## The emergence of superbugs

Antimicrobial resistance (AMR) is already a major driver of mortality and healthcare strain. A landmark analysis estimated 1.27 million deaths attributable to bacterial AMR in 2019, with nearly 5 million deaths associated with AMR (3). GI infections sit at the intersection of community transmission, food systems, and healthcare exposures, making them both a sentinel and a propagator of resistance.

The World Health Organization’s 2024 Bacterial Priority Pathogens List highlights several enteric organisms of high concern, including fluoroquinolone-resistant Salmonella Typhi, Shigella spp., and non−typhoidal Salmonella, as well as third−generation cephalosporin− and carbapenem−resistant Enterobacterales (23). These categories map directly onto clinical dilemmas in dysentery, enteric fever, and healthcare-associated diarrhea.

In the United States, CDC has reported an increase in extensively drug−resistant (XDR) Shigella, defined by resistance to azithromycin, ciprofloxacin, ceftriaxone, trimethoprim-sulfamethoxazole, and ampicillin (24). XDR Shigella highlights how resistance converges with person-to-person transmission in vulnerable networks, increasing the likelihood of outbreaks and limiting outpatient treatment options.

The superbug challenge in GI infections is not only “new antibiotics.” It is also: (i) preventing infections (vaccines, WASH, food safety), (ii) reducing unnecessary antimicrobial exposure, (iii) strengthening surveillance and laboratory capacity, (iv) addressing One Health drivers (including agriculture and environmental reservoirs), and (v) investing in alternatives such as microbiome repair, phage therapy, and anti−virulence strategies.

## What’s up with stewardship?

Stewardship in GI infections has two intertwined domains: antimicrobial stewardship (appropriate selection, dose, route, duration) and diagnostic stewardship (appropriate testing and interpretation). Both are essential because many diarrheal syndromes are viral or self−limited, while indiscriminate antibiotic use increases resistance, harms the microbiome, and raises CDI risk (7, 12).

Key stewardship gaps include: outpatient over-prescribing for uncomplicated diarrhea; misuse of empiric therapy when clinical predictors of invasive bacterial infection are absent; “treating the panel” rather than treating the patient (e.g., unnecessary therapy for certain E. coli pathotypes); and limited access to susceptibility testing as CIDTs displace culture. For H. pylori, stewardship is inseparable from resistance-aware regimen selection and a commitment to test-of-cure (20).

Several levers can accelerate progress. First, guidelines provide syndrome-based frameworks that match testing and therapy to severity and epidemiologic risk (12). Second, coupling multiplex diagnostics with stewardship interventions clinical decision support, structured reporting that flags likely colonization, and reflex culture pathways can reduce inappropriate therapy while preserving time-sensitive treatment. Third, stewardship must incorporate patient-centered outcomes (symptom duration, functional status, recurrence, post-infectious irritable bowel syndrome, and quality of life) alongside traditional endpoints such as length of stay and antibiotic days.

Finally, stewardship is an implementation science problem. The field needs pragmatic trials that evaluate how different report designs, order sets, and care pathways influence decisions in the emergency department, urgent care, and inpatient settings—where most antibiotics for GI syndromes are initiated.

## The microbiome is the future

The microbiome is not merely a “context” for GI infections—it is a mechanistic driver of susceptibility, severity, and recurrence, and a therapeutic target. CDI is the clearest proof of principle: microbiota-based therapies can restore colonization resistance and prevent recurrence (16, 17). Regulatory approval of standardized microbiota-based products for prevention of recurrent CDI marks a turning point, enabling consistent manufacturing, pharmacovigilance, and scalable access (18, 19).

Looking ahead, microbiome science can reshape GI infections in at least four ways. First, prediction: microbial and metabolomic signatures may identify patients at risk of severe disease, complications, post−infectious sequelae, or recurrence. Second, prevention: targeted microbiome modulation may prevent CDI and potentially reduce colonization with resistant Enterobacterales. Third, precision therapy: live biotherapeutic consortia, rational synbiotics, and bacteriophage or CRISPR-based approaches may offer pathogen-specific control with less collateral damage than broad-spectrum antibiotics. Fourth, diagnostics: metagenomic workflows can unify pathogen detection, resistance prediction, and outbreak typing (14, 15).

The grand challenge is moving from association to action: causal inference, mechanistic trials, standardized endpoints, and careful long−term safety monitoring. Equally important is translation—creating interventions that are affordable and deployable in low−resource settings where the burden of enteric disease remains greatest.

## Final remarks

Gastrointestinal infections are simultaneously ancient and emerging: the same syndromes of diarrhea, dysentery, and colitis now unfold in a world of global travel, accelerating resistance, expanding immunosuppression, and rapidly advancing diagnostics and microbiome therapeutics. The next decade’s priorities should include:

- A renewed prevention agenda that integrates vaccines, WASH, and food safety with AMR mitigation.
- Diagnostic strategies that are faster and more informative (including resistance and typing), while minimizing overdiagnosis and inequity.
- Therapeutics that are microbiome-sparing, evidence-based, and tailored to local resistance patterns and patient risk.
- Stewardship frameworks that fuse diagnostic and antimicrobial stewardship, supported by behavioral science and implementation research.
- A translational microbiome pipeline that prioritizes safety, scalability, and rigorous clinical outcomes.

Frontiers in Gastroenterology’s Gastrointestinal Infection section will prioritize work that advances these goals through multidisciplinary research, clinical trials, policy and practice reviews, and innovation in diagnostics, therapeutics, prevention, and the microbiome. The grand challenge is clear: reduce the global burden of GI infections while safeguarding antimicrobials and harnessing modern diagnostics and microbiome science to deliver precision, equity, and durable prevention.
